# Hip Manipulation Increases Electromyography Amplitude and Hip Joint Performance: A Double-Blind Randomized Controlled Study

**DOI:** 10.3390/life14111353

**Published:** 2024-10-23

**Authors:** Rafał Studnicki, Karol Skup, Monika Sochaj, Bartłomiej Niespodziński, Piotr Aschenbrenner, Radosław Laskowski, Piotr Łuczkiewicz

**Affiliations:** 1 Department of Physiotherapy, Medical University of Gdańsk, 7 Dębinki Street, 80-211 Gdańsk, Poland; 2 Student Scientific Circle of Orthopaedic Physiotherapy, 2nd Division of Orthopaedics & Kinetic Organ Traumatology, Medical University of Gdansk, 80-952 Gdansk, Poland; skupkarol@gumed.edu.pl (K.S.); monikasochaj@gumed.edu.pl (M.S.); 3 Department of Biological Foundations of Physical Education, Faculty of Health Sciences and Physical Education, Kazimierz Wielki University, Sportowa 2, 85-091 Bydgoszcz, Poland; bar.niespodzinski@wp.pl; 4 Department of Physical Education, Gdansk University of Physical Education and Sport, 80-336 Gdansk, Poland; piotr.aschenbrenner@awf.gda.pl; 5 Department of Physiology, Gdansk University of Physical Education and Sport, 80-854 Gdansk, Poland; radoslaw.laskowski@awf.gda.pl; 6 2nd Division of Orthopaedics & Kinetic Organ Traumatology, Medical University of Gdańsk, 80-952 Gdansk, Poland; piotr.luczkiewicz@gumed.edu.pl

**Keywords:** musculoskeletal manipulations, hip joint, gluteus medius, superficial electromyography

## Abstract

(1) Background: Activation of the gluteus medius (GM) muscle while minimizing the involvement of the tensor fascia latae (TFL) is crucial in treating many lower limb and lumbar spine injuries. Previous studies have demonstrated the effectiveness of joint manipulations in regulating muscle activity. The main objective of this study was to evaluate the effects of hip joint manipulation (HJM) on the muscle strength and activity (GM and TFL) of hip abductors in asymptomatic young participants. (2) Methods: The study followed a double-blind randomized controlled design. Thirty healthy, physically active women and men, free from spinal and lower limb injuries, voluntarily participated. The participants were allocated to two groups: those allocated to the HJM intervention and those in the control group receiving a sham intervention. They were assessed before and after the intervention using surface electromyography to measure muscle activation (EMG_RMS_) of the GM and TFL during maximal voluntary isometric hip abduction. (3) Results: HJM resulted in a significant increase in EMG_RMS_ amplitude solely within the GM muscle (*p* < 0.01); (4) Conclusions: This study suggests that HJM may increase EMGRMS amplitude in the GM muscle; however, the effects are neither statistically nor clinically significant when compared to the control group for most of the muscles analyzed.

## 1. Introduction

Incorrect biomechanics of the hip joint can result from altered activity of the hip muscles, contributing to various diseases of the lower limbs and lower back [1,2]. The gluteus medius (GM) muscle, the primary abductor of the hip joint, plays a crucial role in stabilizing the pelvis and lower limbs in the transverse and frontal planes during functional activities and gait [3]. Dysfunction of the GM muscle may be associated with pain and functional problems in the hip joint as well as in the lumbar spine [4]. Another muscle that aids in hip joint stability is the tensor fascia latae (TFL). Numerous studies have shown that when the primary muscle responsible for a specific movement weakens, the synergistic muscle compensates, indicating that a weakening GM leads to increased activity and shortening of the TFL. An overactive or overly tense TFL causes increased anterior pelvic tilt, leading to internal hip rotation, which in turn causes incorrect positioning of the hip joint and lumbar spine [5,6]. Evidence suggests that these changes are clinically associated with atrophy or weakness of the GM muscle [7]. Research reports point to various related musculoskeletal conditions, including iliotibial band friction syndrome [8], chronic low back pain [9], groin injuries [10], and the development of degenerative changes in the hip joint [11].

In such situations, a rehabilitation program is typically introduced, featuring exercises such as lunges and bridges, which preferentially activate the gluteal muscles over the TFL [12,13]. Another method for treating musculoskeletal disorders is manual therapy, which includes manipulation [14,15]. Currently, manipulations are used as a form of physiotherapy in conservative management. This technique is defined as a passive, high-velocity, low-amplitude thrust that separates the opposing surfaces of the synovial joint [16]. For any intervention to be therapeutically effective, it must target a biological mechanism through which it can initiate one or more physiological effects [17].

Manipulation, besides its mechanical impact, affects the nerve fibers located in the joint capsule and synovial folds, which act as nociceptors [18]. These manipulation-induced biomechanical changes influence sensory signaling from tissues via mechanoreceptive neuronal terminals [19]. Reduced GM activation can lead to compensatory mechanisms, with the TFL taking over the functions of the primary abductor, masking changes in sensory signaling [20]. Manipulation can reduce these compensatory mechanisms, helping restore the balance of GM muscle activity and improving the functional relationship between GM and TFL [21]. When a muscle is rapidly stretched, as in manipulation, the viscoelastic tissues can influence overall muscle activity [22].

Studies have shown that spinal manipulation can lead to increased maximum voluntary isometric contraction (MVIC) [23,24]. This is potentially associated with transient inhibition of human motoneurons, as evidenced by reduced H-reflex and resting surface electromyography (SEMG) amplitude [25,26]. However, conflicting results exist, such as findings that the quadriceps muscle H-reflex can be unaffected by lumbar manipulation [27]. It is suggested that spinal manipulation alters motor preparation and commands from the supraspinal regions, enhancing control over force production [28].

While many studies have documented the compensatory role of the TFL when the GM weakens, there remains a significant gap in research regarding the effectiveness of manual therapy, specifically hip joint manipulation, in restoring GM function and mitigating TFL overactivity. Understanding these effects could facilitate the identification of therapeutic approaches to enhance clinical outcomes. One interesting method for assessing the impact of manual therapy on muscle function is SEMG, which allows for the evaluation of muscle activity during MVIC. SEMG provides indirect insights into motor unit recruitment and firing rates. Therefore, the aim of this study was to evaluate the effects of hip joint manipulation (HJM) on the muscle strength and activity of hip abductors in asymptomatic young participants by comparing the intervention group receiving HJM against a control group undergoing sham intervention.

## 2. Materials and Methods

This study followed the Consolidated Standards of Reporting Trials (CONSORT) guidelines for reporting randomized experimental studies [29].

### 2.1. Trial Design

A prospective, randomized, double-blind design was used to evaluate the effect of HJM on the strength and activity of the GM and TFL muscles. Participants were randomly allocated into two equal groups: one group received the intervention (manipulation), while the other received a sham treatment. The allocation ratio was 1:1.

### 2.2. Ethical Procedures

The study was approved by the Independent Bioethics Committee for Scientific Research at the Medical University of Gdańsk (Resolution No. NKBBN/866/2022-2023). The study protocol was thoroughly explained to the participants in easy-to-understand language, and written informed consent was obtained from each volunteer, explicitly stating that they were free to withdraw from the study at any time without penalty. The study adhered to the ethical standards for research involving humans as summarized in the Declaration of Helsinki.

### 2.3. Participants

The study was conducted with the following a priori inclusion criteria: (i) healthy men and women; (ii) aged between 19 and 26 years; and (iii) available to participate in all intervention and assessment sessions. The exclusion criteria were: (i) participants with a history of lower limb or lumbar spine surgery; (ii) lower limb injury within the last 6 months; (iii) pain in the ankle, knee, or hip joint; (iv) hypermobility of the ankle joint; (v) rheumatic diseases; (vi) neurological diseases; (vii) neoplastic diseases; (viii) connective tissue diseases; (ix) symptoms of spinal root compression, sciatica, or spinal canal stenosis; and (x) history of manipulation treatment.

**Figure 1:**
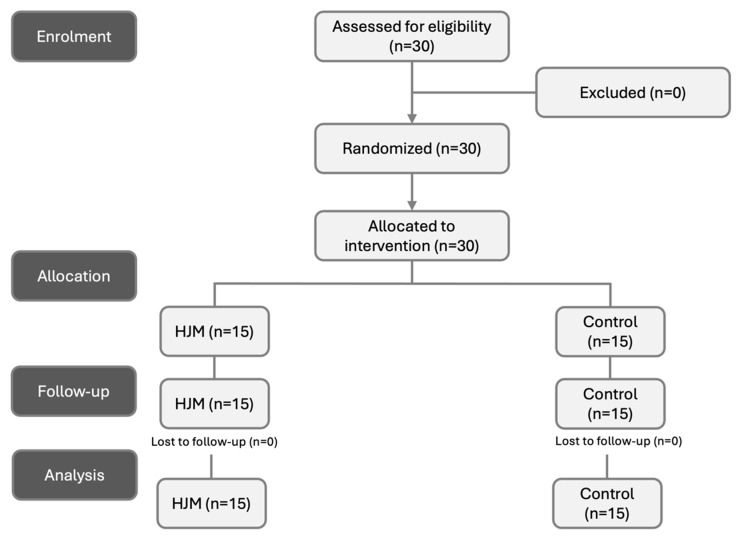
Participant flow across different phases. HJM: Hip Joint Manipulation Group.

The recruitment process involved announcements in the primary intervention area, postings on social media, and direct contacts. Using a simple sampling strategy, 30 healthy men and women expressed interest in volunteering. All these individuals were confirmed to be eligible for the experiment based on predefined criteria. Among the participants, 22 were men and 8 were women. After randomization, they were allocated to either the HJM group or the control group (Figure 1). The characterization of the participants is shown in Table 1.

### 2.4. Interventions

Participants in the intervention group (i.e., HJM) received high-velocity, low-amplitude (HVLA), and short-duration traction manipulation. The intervention was conducted with a 48-hour rest period, taking into account any physical exertion by the participants. The interventions took place in the morning in a dedicated quiet room where only the participant and the practitioner were present. The room was maintained at 22 °C with a relative humidity of 50%. The intervention was administered as follows: Participants were informed about the procedure. Each participant lay in a supine position with 30° flexion in the hip joint and their arms placed along the trunk. The therapist positioned himself on the lateral side of the participant, wrapping both hands around the proximal thigh as close to the hip joint as possible. The therapist then performed a thigh movement, moving it laterally, perpendicular to the axis of the lower limb, until the maximum tension of the tissues in the hip joint was achieved. From this point, a distance of 1 cm was measured between the patient’s thigh and the therapist’s torso to standardize the repeatability for subsequent interventions. At this point, the therapist made sure that the participant did not feel any discomfort and then performed a 1-s lateral manipulation. This technique was performed once for each participant. Each manipulation was performed by a physiotherapist specializing in manual therapy with 25 years of experience.

Participants in the control group received a sham intervention. The procedure mirrored the HVLA technique, but without inducing tissue tension during the lateral movement of the thigh. Upon reaching the desired position, the therapist used their torso to block further lateral displacement of the thigh, preventing any increase in tissue tension in the hip joint. The therapist then ensured the participant felt no discomfort and performed a movement that imitated manipulation without increasing tissue tension [30].

### 2.5. Outcomes

The study took place at the Physical Exercise Laboratory of the Department of Biomechanics and Sports Engineering, Academy of Physical Education in Gdańsk. To ensure the reliability of the therapeutic assessment, volunteers underwent testing twice by the same physiotherapists.

Before and after the intervention, participants underwent testing to measure muscle strength in the hip joint and muscular activity using SEMG. The evaluations were carried out by experienced assessors who were familiar with and had prior experience using the Biodex System 4 and EMG, the necessary instruments for these assessments.

### 2.6. Muscle Strength Assessment

**Figure 2:**
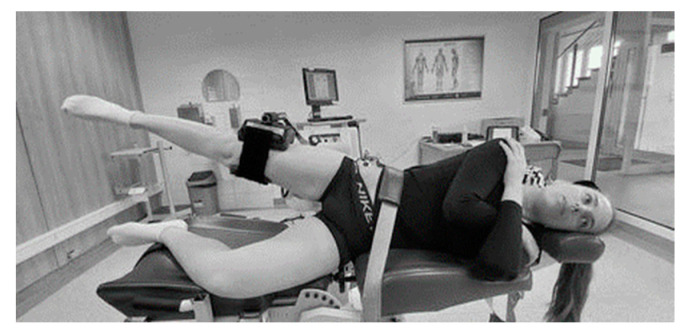
The position of volunteers during muscle strength and electromyographic assessment.

Muscle strength of the hip joint abductors was assessed using the Biodex System 4 (Biodex Medical Systems Inc., Shirley, NY, USA). Participants were positioned according to the manufacturer’s guidelines: lying on their non-tested side, with the tested lower limb abducted 45° at the hip joint (neutral in sagittal and transverse planes), and the knee extended (Figure 2). The tested lower limb was secured to the device’s arm using leather straps, and the device’s rotation shaft was aligned with the anatomical axis of the hip joint for movement in the frontal plane. The participant’s pelvis was stabilized with additional leather straps. In this position, each participant performed three 5-s maximal voluntary isometric contractions (MVIC). There was a 30-s break between each repetition, and participants were verbally encouraged to exert maximum effort during each contraction. The highest peak torque (Nm) from the three attempts was selected for further analysis. Peak torque was also normalized to each participant’s body mass (Nm × kg^−1^).

### 2.7. Assessment of Muscle Activity

SEMG data were recorded from the tensor fasciae latae (TFL) and gluteus medius (GM) during maximum voluntary isometric contraction (MVIC) of hip abduction. The SEMG data were collected and amplified with a differential gain of 500 utilizing the TeleMyo DTS system (Noraxon, Scottsdale, AZ, USA) in conjunction with 1-cm^2^ Ag/AgCl surface electrodes (Sorimex, Toruń, Poland). The SEMG signals underwent band-pass filtering within the frequency range of 15 to 500 Hz, and they were sampled at a rate of 1500 Hz with 16-bit resolution through an analog-to-digital converter. Following this, the SEMG data were stored and subjected to additional analysis using MyoResearch 2.8 software (Noraxon). The placement of electrodes and skin preparation processes—including shaving, abrasion, and alcohol cleaning—were performed in accordance with SENIAM guidelines. The signal processing comprised full rectification and subsequent smoothing using the root mean square (EMG_RMS_) technique with a moving time window of 300 ms. The SEMG results analyzed included the mean and maximum amplitude of EMG_RMS_ (measured in µV) and the median frequency of the power spectrum of the raw SEMG signal (EMG_MED_, in Hz).

### 2.8. Sample Size

The study’s sample size was calculated using G*Power (version 3.1.9.6, Universität Düsseldorf, Düsseldorf, Germany). The calculations were based on ANOVA repeated measures within-between interaction, assuming a moderate effect size of 0.5, a power of 0.85, two groups, and three measurements, resulting in a suggested sample size of 10 participants.

### 2.9. Randomization

The simple randomization process assigned participants identification numbers in a 1:1 ratio using Research Randomizer software (Versja 4.0). Allocation concealment was ensured by randomizing and assigning participants before the initial evaluation. There were no changes in group assignments for any participants.

### 2.10. Blinding

The participants were blinded to the intervention, ensuring they did not observe other participants, and sham therapy was conducted in the control group to maintain uniform conditions across all groups. The evaluators who conducted the assessments were also blinded to the participants’ group allocations.

### 2.11. Statistical Methods

The study presented descriptive statistics, including means and standard deviations. Before proceeding with inferential statistics, the normality of the sample was assessed per each outcome, and confirmation was sought through the Kolmogorov–Smirnov test (*p* > 0.05). The Kolmogorov–Smirnov test was conducted by comparing the empirical cumulative distribution function (ECDF) of a sample with a theoretical cumulative distribution function or another sample’s ECDF to assess whether they are drawn from the same distribution. Similarly, verification of the assumption of homogeneity was conducted using Levene’s test (*p* > 0.05). A mixed ANOVA was implemented to test the interactions between time (the three assessments) and groups (experimental and control), thus providing information allowing to examine differences between groups and changes over time or conditions per outcome. In the mixed ANOVA, partial eta squared ($\eta_{p}^{2}$) was calculated as a measure of effect size. Additionally, post-hoc comparisons were conducted using the Bonferroni test to evaluate differences between assessment time points and to compare groups at each assessment point. All statistical analyses were performed using SPSS software (IBM SPSS Statistics, Version 29.0.2.0; Armonk, NY, USA: IBM Corp.), with a predetermined significance level of *p* < 0.05.

## 3. Results

**Figure 3:**
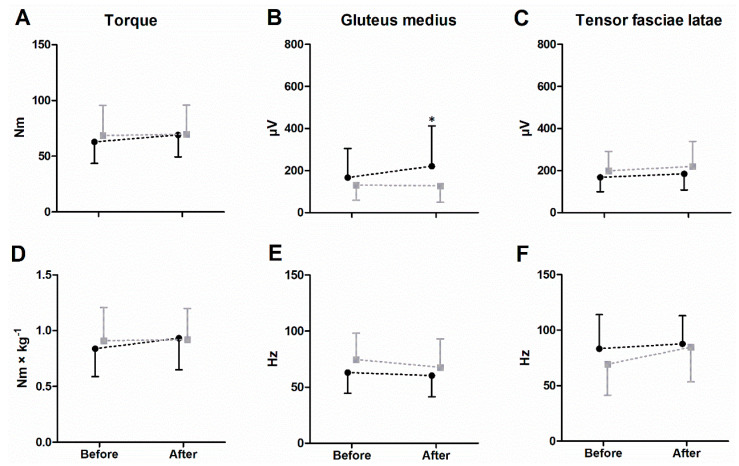
Descriptive statistics of the outcomes collected during both the pre- and post-intervention assessments for both groups. Absolute (**A**) and relative (**D**) peak torque; mean EMG_RMS_ amplitude (**B**,**E**) and median frequency of power spectrum (**C**,**F**). * Within-group significant variations (post-pre) at *p* ≤ 0.05. Black: experimental group; Grey: control group.

Figure 3 shows the average and standard deviation of the outcomes collected during both the pre- and post-intervention assessments for both groups. In the case of peak torque, there was a significant main effect of repeated measures (time) factor in both absolute and normalized values (*p* ≤ 0.05), with results increasing by up to 12% following the intervention. Post-hoc tests indicated that this increase was primarily observed in the intervention group (HJM).

Considering SEMG results after the hip manipulation, there were significant repeated measures (time) effects (*p* ≤ 0.05) for the TFL, where after the manipulation the EMG_MED_ increased by 12.9% regardless of the group (Table 2). The same effect was also observed in GM, where EMG_RMS_ amplitude also increased, but the significant interaction showed that was due to the increase only in the intervention group (Figure 3).

## 4. Discussion

The current study showed an increase in EMG_RMS_ in the GM muscle after the HJM; however, no other significant results were observed. Additionally, the findings do not provide evidence of between-group differences, suggesting that HJM may not have a significant effect on muscle strength and activity.

A previous study [31] revealed that high-velocity low-amplitude hip mobilization in participants with knee dysfunction resulted in increased MVIC of hip extension but not abduction. Similar SEMG outcomes to the current study were observed when manipulating the spine in participants with lower back pain, where the erector spinae muscles increased their activation during MVIC [32]. The authors [32] suggest that this observed outcome could be attributed to mechanical stimulation of the somatosensory system, inhibition of nociception, improvement in muscle functional ability, and/or enhanced range of motion. Conversely, non-thrust grade IV inferior hip joint mobilization elicited a 17% increase in hip MVIC abduction [33]. The contrasting results can be caused by the variability in individual responses to manual therapies, which may lead to inconsistent outcomes across participants, diluting the overall statistical effect. Moreover, only one session was conducted, thus possibly the duration and frequency of the therapy sessions administered might not have been optimal to induce measurable changes in MVIC strength. Furthermore, biomechanical and neurophysiological mechanisms underlying joint mobilization effects on muscle strength remain complex, which requires further research to clarify their full impact on MVIC.

While both the GM and TFL muscles contribute to hip abduction, changes in SEMG amplitude were observed only in the GM muscle. This suggests that the applied manipulation intervention primarily influenced the function of the main hip abductor, the GM muscle. Previous research [18] has shown that during side-lying hip abduction, similar to the testing in the current study, GM activity is approximately 34% higher than TFL activity. Furthermore, this position elicited one of the highest activations (56% relative to MVIC) compared to many other lower limb exercises [6]. Therefore, any alterations in hip abductor muscle activity should primarily manifest in the GM muscle.

One factor that could have influenced the observed results was the position of hip rotation. While the leather straps restricted thigh movement, it is possible that some rotational torque was generated by the abductor muscles. Previous studies [34] have demonstrated that in a similar side-lying hip abduction position, approximately 30° against gravity, GM and TFL muscle activity varies depending on thigh rotation. Medial rotation increases GM muscle activity, whereas lateral rotation increases TFL activity. However, during MVIC testing, the position of the hip joint is irrelevant [35], and this should also be considered in the current study.

The observed increase in EMG_RMS_ of the gluteus medius muscle following HJM may also be related to an activation pattern that may be linked to specific mechanoreceptor activity and sensory feedback loops involved in proprioception and neuromuscular control. Mechanoreceptors located within the joint capsule, ligaments, and surrounding musculature play a crucial role in sensing mechanical changes during manipulation, contributing to the modulation of muscle activity through reflex pathways that integrate sensory input with motor output [36]. The enhanced activation of the GM muscle, noted in this study, could thus be interpreted as a reflection of heightened proprioceptive input and neural engagement following HJM, which may promote motor unit recruitment and improve overall muscle coordination [37].

The lack of significant variation in torque and EMG amplitudes in the tensor fasciae latae (TFL) muscle following hip joint manipulation, compared to a control group receiving the sham intervention, may be related to the stability of torque production, and EMG readings suggest that the neuromuscular control system, particularly the motor unit recruitment patterns, remained unaltered by the intervention [38]. This could be due to the inherent properties of the TFL, which may exhibit a relatively high threshold for change in response to mechanical manipulation. Additionally, the specificity of hip joint manipulation might not have sufficiently influenced the neuromuscular pathways associated with TFL activation, as these pathways are predominantly modulated by central nervous system mechanisms, which may not be directly engaged by the manipulation itself [18]. Furthermore, factors such as muscle fatigue, pre-existing tension, or adaptive neural pathways could have maintained consistent EMG amplitudes despite the intervention. Lastly, the sham intervention group may have experienced similar psychological and physiological effects due to participant expectation and attention, potentially leading to a placebo effect that masked any differences between the two groups. Thus, the interplay of these physiological and psychological factors could elucidate the observed stability in torque and EMG amplitudes in the TFL across both experimental conditions.

The current study verified increased EMG_RMS_ in the GM muscle post-HJM but lacked significant findings elsewhere and between-group differences, suggesting a limited impact on overall muscle strength and activity. One key limitation of this study is the short duration of the manipulation intervention, consisting of only a single session, which may have been insufficient to elicit meaningful changes in muscle strength and activation. To better understand the cumulative effects of hip joint mobilization, future research should investigate multiple sessions over an extended period. Furthermore, individual variability in responses to manual therapies can significantly influence outcomes; variations in participants’ anatomy, neuromuscular control, and baseline muscle activation may lead to inconsistent results that dilute statistical significance. This highlights the necessity for personalized treatment approaches tailored to individual needs. Additionally, the assessment conducted immediately post-manipulation may overlook potential longer-term adaptations, indicating a need for future studies to evaluate muscle activation at various intervals after treatment. Understanding these factors is crucial for a comprehensive interpretation of the effects of hip joint manipulation on muscle function. The specific increase in GM activation relative to the TFL, despite both muscles contributing to hip abduction, emphasizes the need to further explore the biomechanical and neurophysiological mechanisms underlying joint mobilization effects on muscle strength. Lastly, limitations such as the exclusive reliance on immediate post-manipulation assessments and the random selection of the examined limb using a “coin toss” method complicate the interpretation of results concerning dominant limb effects.

Also, new research can take advantage of distributed control systems and data-driven learning. For example, a previous study [39] outlines a distributed control architecture for electrohydraulic humanoid robots that emulates the human nervous system, emphasizing real-time capabilities and adaptable software that functions in both centralized and decentralized modes. This adaptability is crucial in biomechanics research, where the complexity of human motion requires a control system that can dynamically respond to changes in the environment and system state. Another example [40] introduces an adaptive H∞ control scheme utilizing data-driven learning techniques to accurately estimate unknown system dynamics without the typical errors associated with neural network approximations. By integrating these data-driven methodologies, researchers can develop biomechanical models that continuously learn and adapt in real time, thereby optimizing rehabilitation protocols in physiotherapy. For instance, data-driven techniques could be employed to analyze patient feedback and adjust treatment plans accordingly, facilitating a more personalized rehabilitation experience. This approach not only enhances the precision of therapeutic interventions but also contributes to a deeper understanding of sensorimotor control, ultimately bridging the gap between biomechanics and physiotherapy and improving patient outcomes.

While the current study suggests limited immediate effects of HJM on overall muscle strength and activity, the observed increase in GM muscle EMG_RMS_ indicates a potential benefit for targeting specific muscles involved in hip abduction. Clinicians should consider HJM as a targeted intervention for enhancing GM muscle activation in conditions where strengthening this muscle is beneficial. However, due to the variability in individual responses and the unclear duration of these effects post-intervention, therapists should adapt treatment protocols to account for patient-specific factors and monitor long-term outcomes.

## 5. Conclusions

The findings revealed an increase in EMG_RMS_ in the GM muscle following HJM, suggesting a potential benefit in enhancing its activation during hip abduction tasks. However, broader implications on overall muscle strength and activity were limited, as no significant between-group differences were observed. These outcomes underline the variability in individual responses to manual therapies like HJM, which may contribute to inconsistent results across participants. Future research should explore the biomechanical and neurophysiological mechanisms underlying the effects of HJM in greater depth, with the goal of better understanding the processes and outcomes of these interventions. Clinically, our results suggest that HJM may have some relevance for the GM muscle, though it may not be significant in other muscles. However, this evidence should be interpreted with caution by practitioners, considering the specific needs of each patient.

## Figures and Tables

**Table 1:** Demographic characteristics of the participants.

	HJM (*n* = 15)	Control (*n* = 15)	Overall (*n* = 30)
Age (years)	21.9 ± 1.69	22.1 ± 1.8	22.0 ± 1.7
Height (cm)	178.3 ± 9.68	181.5 ± 9.5	179.9 ± 9.6
Body mass (kg)	74.4 ± 11.6	76.6 ± 16.2	75.5 ± 14.1

HJM: Hip Joint Manipulation Group.

**Table 2:** Two-way ANOVA with repeated measures (2 × 2) of peak torque and surface electromyographic outcome after hip manipulation during hip abduction.

Variable		Factor	F (df)	*p*	η^2^	Post-Hoc
**Abduction torque**						
**Peak torque**	Absolute	GR	0.13 (1,28)	0.72	0.01	
		RM	4.37 (1,28)	0.05 *	0.14	(I) < (II)
		GR × RM	2.01 (1,28)	0.17	0.07	
	Relative to body mass	GR	0.08 (1,28)	0.77	0.01	
		RM	4.22 (1,28)	0.05 *	0.13	(I) < (II)
		GR × RM	2.98 (1,28)	0.09	0.10	
**Surface electromyography**						
**Tensor facia latae**	SEMG Amplitude	GR	1.14 (1,28)	0.29	0.04	
		RM	2.16 (1,28)	0.15	0.07	
		GR × RM	0.03 (1,28)	0.87	0.01	
	SEMG Frequency	GR	0.73 (1,28)	0.40	0.03	
		RM	6.97 (1,28)	0.01 *	0.20	(I) < (II)
		GR × RM	2.14 (1,28)	0.15	0.07	
**Gluteus medius**	SEMG Amplitude	GR	1.98 (1,28)	0.17	0.07	
		RM	5.93 (1,28)	0.02 *	0.18	(I) < (II)
		GR × RM	8.13 (1,28)	0.01 *	0.23	INT(I) < INT(II)
	SEMG Frequency	GR	1.66 (1,28)	0.21	0.06	
		RM	2.49 (1,28)	0.13	0.08	
		GR × RM	0.47 (1,28)	0.49	0.02	

SEMG—surface electromyography, GR—group; RM—repeated measure (time); INT—intervention; (I)—before measure; (II)—after measure; * significance at *p* ≤ 0.05.

## Data Availability

The data can be provided upon a reasonable request to the corresponding author.
